# Germline Compound Heterozygous Variants Identified in the *STXBP2* Gene Leading to a Familial Hemophagocytic Lymphohistiocytosis Type 5: A Case Report

**DOI:** 10.3389/fped.2021.633996

**Published:** 2021-06-24

**Authors:** Vera Maria Dantas, Cassandra Teixeira Valle, Roberta Piccin de Oliveira, Mylena Taíse Azevedo L. Bezerra, Cleia Teixeira do Amaral, Raissa Anielle S. Brandão, Jussara M. Cerqueira Maia, Tirzah Braz Petta

**Affiliations:** ^1^Department of Pediatrics, Pediatric Immunology Division of Onofre Lopes University Hospital, Federal University of Rio Grande do Norte, Natal, Brazil; ^2^Pediatric Hematology Division of Onofre Lopes University Hospital, Federal University of Rio Grande do Norte, Natal, Brazil; ^3^Pediatric Allergy-Immunology Division, Onofre Lopes University Hospital, Federal University of Rio Grande do Norte, Natal, Brazil; ^4^Pediatric Infectiology Division, Onofre Lopes University Hospital, Federal University of Rio Grande do Norte, Natal, Brazil; ^5^Pediatric Pneumology Division, Onofre Lopes University Hospital, Federal University of Rio Grande do Norte, Natal, Brazil; ^6^Department of Pediatrics, Pediatric Gastroenterology Division of Onofre Lopes University Hospital, Federal University of Rio Grande do Norte, Natal, Brazil; ^7^Department of Cellular Biology and Genetics, Federal University of Rio Grande do Norte, Natal, Brazil

**Keywords:** pancytopenia, hepatosplenomegaly, hyperferritinemia, STXBP2, familial hemophagocytic lymphohistiocytosis type 5

## Abstract

Familial hemophagocytic lymphohistiocytosis (FHL) is a rare, potentially fatal autosomal-recessive immunodeficiency, and *STXBP2* mutations have been associated with FHL type 5 (FHL-5). Here, we report a case of a 2-year-old boy who presented with recurrent fever, hepatosplenomegaly, pancytopenia, hyperferritinemia, and hypofibrinogenemia since 4 months of age. His genetic analysis revealed a compound heterozygosity of the *STXBP2* gene with a described pathogenic mutation, c.1247-1G>C (splicing acceptor site), harbored by his father and a likely pathogenic variant of uncertain significance (VUS), c.704G>A (p.Arg235Gln), harbored by his mother. He was diagnosed as compound heterozygous for FHL-5 and was treated with the HLH-2004 protocol. Since treatment, this patient has been in remission, and he is being evaluated for a hematopoietic stem cell transplantation (HSCT).

## Introduction

Familial hemophagocytic lymphohistiocytosis (FHL) is a rare disease with an autosomal-recessive inheritance pattern. It is caused by defects in immune regulation, such as mutations in genes controlling cytotoxicity and the killing effect of natural killer (NK) and T cells (1, 2). FHL is included in inherited hemophagocytic lymphohistiocytosis disorders, also known as primary hemophagocytic lymphohistiocytosis (HLH) (2).

There are several mutations associated with congenital immunodeficiency syndromes that can lead to episodes of immune dysregulation and HLH: Chédiak–Higashi syndrome (*LYST*), Griscelli syndrome type 2 (*RAB27A*), Hermansky–Pudlak syndrome (*AP3B1*), and X-linked lymphoproliferative syndrome (XLP)-1 (*SH2D1A*) and XLP-2 (*XIAP*) (3).

Secondary HLH includes patients without a known familial mutation. These patients can also develop acute HLH in the context of infections, malignancies, autoinflammatory or metabolic diseases, and acquired immunodeficiencies (4). Viral infections are frequently implicated in the onset of active HLH episodes, both in primary, genetic HLH, as well as in the secondary acquired form. The most common infections are those of herpes viruses, such as Epstein–Barr virus and cytomegalovirus (4).

According to Janka and Stadt, as cited by George (3), FHL syndromes are subclassified into five subtypes (FHL-1 to FHL-5) based on the functional protein anomalies and the prerequisite genetic mutations. Viñas-Giménez et al. (5) related four genes (*PRF1, UNC13D, STX11*, and *STXBP2*) in the 9q21.3–22 region that were identified as possible causes of FHL-2, FHL-3, FHL-4, and FHL-5, respectively. The mutated gene for the FHL-1 subtype is currently undefined. Sieni et al. (6) have reported that variants in these four genes comprise 240 missense, 69 frameshift, 51 non-sense, 51 splicing, 10 in-frame indel, 7 deep intronic, and 5 large rearrangement variants. FHL-5 is caused by homozygous or compound heterozygous mutations in the *STXBP2* gene, which has 19 exons and is located in the 19p13 region. This gene encodes the 593-amino acid protein Munc18-2, and its functional alterations may impair the secretion of cytotoxic granules by NK cells (7).

It is estimated that the incidence of FHL is ~0.12–0.15 per 100,000 children per year, and FHL-5 only accounts for 10% of all cases of FHL (8). Generally, the first episodes of the disease occur during infancy, with a peak incidence between 1 and 6 months of age (6). Clinical manifestations of HLH are mainly a result of tissue infiltration by T cells and macrophages as well as an inappropriate pro-inflammatory cytokine release (4). The clinical findings are fever, hepatosplenomegaly, cytopenia, hypofibrinogenemia, hypertriglyceridemia, hyperferritinemia, less frequently, and central nervous system involvement (2, 4).

HLH, whether primary or secondary, can have a fulminant and fatal course or, in some circumstances, may have a non-severe picture with a transient course. Treatment of HLH should be guided by both the severity of the disease and its underlying etiology (3).

In 1994, the Histiocyte Society proposed the first protocol for the treatment of HLH (HLH-94). In 2004, a revised protocol (HLH-2004) was proposed (2). These protocols have greatly improved the outcomes of this frequently life-threatening disease. Both protocols are primarily based on the administration of steroids and etoposide to control the inflammation and its end-organ damage. However, in the case of relapse or that with primary etiology, hematopoietic stem cell transplantation (HSCT) may be required (2, 3).

Herein, we report a case of a patient diagnosed with FHL-5 and canonical phenotype found to be compound heterozygous in the *STXBP2* gene of one known pathogenic mutation and one variant of uncertain significance (VUS) not previously associated with FHL.

## Case Presentation

A 2-year-old boy with recurrent hospitalizations since 4 months of age was admitted with fever, diarrhea, and abdominal distension. His weight and height were in the 50th and fifth percentiles, respectively. His physical examination was noticeable for splenomegaly 8 cm below the left costal margin and hepatomegaly 4 cm below the right costal margin, but there was no palpable lymphadenopathy. His laboratory workup revealed pancytopenia, hypofibrinogenemia, and hyperferritinemia (Table 1). Serological investigations for rubella, cytomegalovirus, herpes simplex virus, hepatitis B, human immunodeficiency virus, toxoplasmosis, visceral leishmaniosis, and autoimmune diseases were negative. His immunologic evaluation showed normal immunoglobulin levels (IgG, IgM, IgA, and IgE) and normal lymphocyte subset counts (CD3, CD4, CD8, CD19, and NK cells). An estimate of neutrophil function using the dihydrorhodamine test was normal and therefore not suggestive of chronic granulomatous disease (CGD). Bone marrow aspiration and biopsy did not show hemophagocytic pictures, malignancy, or infection. Of note is that the patient's parents were not consanguineous, and there was no family history of similar presentations, including in two maternal half-siblings, a 10-year-old boy, and a 13-year-old girl.

**Table 1:** Patient's clinical laboratory findings and HLH diagnostic criteria.

**Findings**	**HLH-2004 diagnostic criteria (at least five out of eight main features)**
Fever^a^	>37°C
Hepatosplenomegaly^a^	Radiographic or physical exam evidence
Cytopenias^a^	2 or 3 hematopoietic lineages
Hemoglobin: 4.7 g/dl	<9 g/dl
Platelets: 28 × 10^9^/L	<100 × 10^9^/L
Neutrophils: 0.48 × 10^9^/L	<1.0 × 10^9^/L
Triglycerides: 210 mg/dl	≥265 mg/dl
Fibrinogen^a^: 1.49 g/L	≤1.5 g/L
Ferritin^a^: 1,160 μg/L	≥500 μg/L
LDH: 793 U/L	≥500 U/L
ALT: 25 U/L	≥100 U/L
AST: 18 U/L	≥100 U/L
Bilirubin: 17.45 μmol/L	≥34 μmol/L
Hemophagocytosis: absent	Present in bone marrow or other
Decreased NK cell activity: –	<10% activity by flow cytometric assay
Elevated sCD25: –	>2,400 U/ml
CSF cells: –	≥5/μL
CSF protein: –	≥0.5 g/L

*ALT, alanine transaminase; AST, asparate aminotransferase; CSF, cerebrospinal fluid; HLH, hemophagocytic lymphohistiocytosis; LDH, lactate dehydrogenase; NK, natural killer; sCD25, soluble CD25*.

a *Patient diagnostic criteria*.

**Figure 1:**
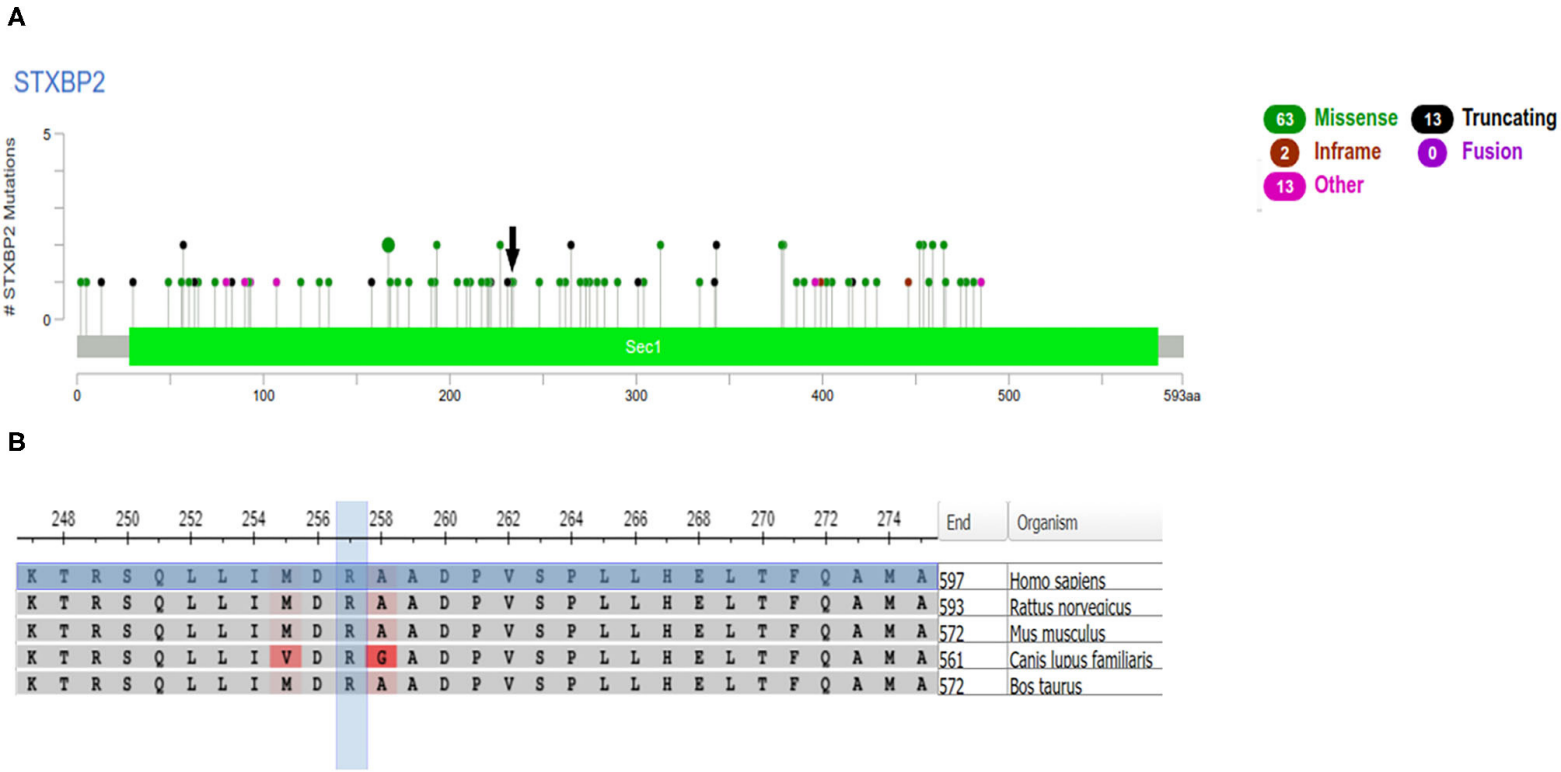
**(A)** Pathogenic variant and a variant of uncertain significance (VUS) for the *STXBP2* gene from ClinVar. The pathogenic variant c.1247-1G>C found in the patient is in a splicing site and therefore is not illustrated in this figure (variation ID: 330555; dbSNP: rs140148806). The VUS found in the patient is indicated by a *black arrow* (c.704>A; p. Arg235gln). There are 10 pathogenic variants and 81 VUS described for *STXBP2* in ClinVar, as shown in the figure. The color legend is indicated in the *corner of the figure*. **(B)** Protein alignment for the residue Arg235 (VUS) among five organisms: *Homo sapiens, Rattus norvegicus, Mus musculus, Canis lupus familiaris*, and *Bos taurus*. Conservation of the arginine (R) is highlighted in *blue*. Alignment position among the five species: 257; sequence position: 235.

The patient was empirically treated with corticosteroids, intravenous immunoglobulins, and antibiotics. This resulted in gradual clinical and laboratorial improvement over the following 3 weeks. The patient had recurrent similar presentations over the following year, but was always responsive to steroids. Then, primary immunodeficiency next-generation sequencing panels of 207 genes (Invitae, San Francisco, CA, USA) were performed on the blood of the patient and his parents. This revealed that the patient had biallelic mutations in the *STXBP2* gene: one pathogenic variant, c.1247-1G>C (splicing acceptor site), and one VUS, c.704G>A (p.Arg235Gln) (Figure 1). Genetic analyses of the patient's parents showed that both were heterozygous for the *STXBP2* gene; the father harbored a pathogenic mutation, c.1247-1G>C (splicing acceptor site), and the mother harbored a VUS, c.704G>A (p.Arg235Gln).

**Figure 2:**
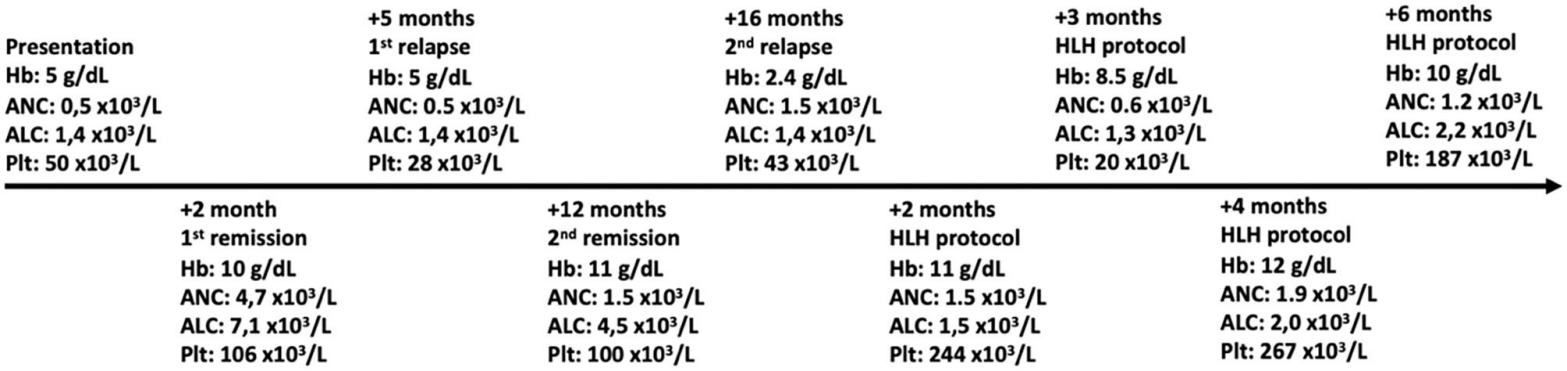
Peripheral blood counts over time. Hb, hemoglobin; ANC, absolute neutrophil count; ALC, absolute lymphocyte count; Plt, platelet count.

The patient was diagnosed with FHL-5 and was started on treatment with the HLH-2004 protocol. Since treatment with this protocol, the patient has been in remission (Figure 2). His family states that he is improving and has had only one relapse of disease (which was associated with the public health service's lack of one of the protocol medications for 2 months). The patient is currently under evaluation for HSCT.

## Discussion

In this case report, we describe a 2-year-old boy from Brazil with five of the eight clinical laboratory criteria for hemophagocytic lymphohistiocytosis (2). He presented with recurrent fever, hepatosplenomegaly, pancytopenia, hypofibrinogenemia, and hyperferritinemia (Table 1) and intercalated with short remission periods after empirical treatment with corticosteroids (Figure 2). Of note is that the bone marrow aspirate did not reveal hemophagocytosis, but this does not rule out the diagnosis of HLH (6).

HLH is often triggered by viral infection in a genetically predisposed child (2). but an extensive viral workup was negative, as described above. Visceral leishmaniasis is a differential diagnosis of HLH (9). particularly in a patient's endemic area, but this was ruled out by a normal rK39 (a sensitivity test) (10) and the bone marrow evaluation.

Primary immunodeficiencies that confer impaired killing of infectious pathogens can cause patients to display the HLH phenotype (e.g., the inappropriate cytokine release in patients with CGD) (11). However, an evaluation for primary immunodeficiencies (including CGD) was negative, as reported above.

The child presented a molecular diagnosis consistent with HLH-5 (MedGen ID: 416514), with heterozygous compound mutations in *STXBP2*, the pathogenic variant c.1247-1G>C, and the VUS c.704G>A (p.Arg235Gln) (Figure 1).

*STXBP2* encodes for the 593-amino acid protein Munc18-2, whose deficiency causes familial hemophagocytic lymphohistiocytosis type 5 (OMIM: 613101) and impairs cytotoxic granule exocytosis by NK cells (7, 12). Studies have reported that individuals with HLH may be compound heterozygotes and may also have monoallelic mutations of known familial HLH genes (13). According to Spessott et al., as cited by Chinn (14), the possibility that some of these monogenic variants may cause dominant-negative loss of function of the corresponding protein cannot be fully excluded.

The patient's reported *STXBP2* VUS c.704G>A (p.Arg235Gln) is presented in a population database (rs757488006, 0.002%; ExAC) and the Invitae database. A VUS in the same amino acid has been reported previously, c.704G>C p.Arg235Pro, with a high likelihood score for pathogenicity (5). Pathogenicity prediction algorithms are controversial for the impact of this missense change (SIFT: “Deleterious”; PolyPhen-2: “Benign”; Align-GVGD: “Class C0”). We have applied a decision tree based on a machine learning technique for data classification, and the algorithm predicted the VUS as pathogenic or damaging (15).

Studies of FHL-5 patients with biallelic mutations in *STXBP2*, homozygous or compound heterozygous mutations, and one allele carrying an exon 15 c.1247-1G>C splice-site mutation showed later-onset, long-lasting remission and mild relapses (16, 17). This supports the assumption of a residual function of exon 15 splice-site mutations with a less complete or partial defect in this pathway. This could explain why our patient survived recurrent disease flare-ups that transiently responded to therapy with only steroids.

The FHL-5 phenotype of this patient was associated with the compound heterozygosity of one known pathogenic *STXBP2* mutation and the VUS c.704>A (p.Arg235gln), highlighting the possible association of this VUS with FHL-5.

## Data Availability Statement

The original contributions presented in the study are included in the article /supplementary material, further inquiries can be directed to the corresponding author/s.

## Ethics Statement

Ethical review and approval was not required for the study on human participants in accordance with the local legislation and institutional requirements. Written informed consent to participate in this study was provided by the participants' legal guardian/next of kin. Written informed consent was obtained from the individual(s), and minor(s)' legal guardian/next of kin, for the publication of any potentially identifiable images or data included in this article.

## Author Contributions

VD provided clinical expertise and conceived and drafted the manuscript. CV provided clinical expertise and provided patient care. CA, RB, and RO collected the case history and additional clinical data. MB and JC provided patient care. TP interpreted genetic data and assisted with preparing the figures and supervising the project. All authors were involved in the critical revision of the manuscript.

## Conflict of Interest

The authors declare that the research was conducted in the absence of any commercial or financial relationships that could be construed as a potential conflict of interest.
